# Plant peroxisomal solute transporter proteins

**DOI:** 10.1111/jipb.12790

**Published:** 2019-04-16

**Authors:** Lennart Charton, Anastasija Plett, Nicole Linka

**Affiliations:** ^1^ Institute for Plant Biochemistry and Cluster of Excellence on Plant Sciences (CEPLAS) Heinrich Heine University Universitätsstrasse 1 40225 Düsseldorf Germany

## Abstract

Plant peroxisomes are unique subcellular organelles which play an indispensable role in several key metabolic pathways, including fatty acid β‐oxidation, photorespiration, and degradation of reactive oxygen species. The compartmentalization of metabolic pathways into peroxisomes is a strategy for organizing the metabolic network and improving pathway efficiency. An important prerequisite, however, is the exchange of metabolites between peroxisomes and other cell compartments. Since the first studies in the 1970s scientists contributed to understanding how solutes enter or leave this organelle. This review gives an overview about our current knowledge of the solute permeability of peroxisomal membranes described in plants, yeast, mammals and other eukaryotes. In general, peroxisomes contain in their bilayer membrane specific transporters for hydrophobic fatty acids (ABC transporter) and large cofactor molecules (carrier for ATP, NAD and CoA). Smaller solutes with molecular masses below 300–400 Da, like the organic acids malate, oxaloacetate, and 2‐oxoglutarate, are shuttled via non‐selective channels across the peroxisomal membrane. In comparison to yeast, human, mammals and other eukaryotes, the function of these known peroxisomal transporters and channels in plants are discussed in this review.

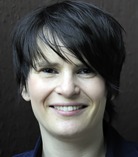

Nicole Linka

**Edited by:** Jianping Hu, Michigan State University, USA

## INTRODUCTION

Peroxisomes are surrounded by a single membrane which functions as a permeability barrier to solutes by forming a confined compartment for peroxisomal metabolism. To connect peroxisomes to the metabolic network of the cell, the peroxisomal bilayer membrane embeds different transport proteins allowing the passage of a variety of solutes (Rottensteiner and Theodoulou 2006; Antonenkov and Hiltunen 2012; Linka and Theodoulou 2013). Since peroxisomes are highly metabolically active, a massive transfer of metabolites across the peroxisomal membrane has to take place (Antonenkov and Hiltunen 2012; Linka and Esser 2012; Linka and Theodoulou 2013). Recent progress defines the permeability of the peroxisomal membrane to solutes into two types of transport proteins: non‐selective pore‐forming channels facilitate the passage of small solutes down the electrochemical gradient, like the β‐barrel porins found in the outer membrane of gram‐negative bacteria, mitochondria and plastids (Antonenkov and Hiltunen 2012); specific transporters mediate the passive flux of larger molecules in a uniport or antiport mechanism, typical for the inner mitochondrial and envelope membrane (Linka and Esser 2012; Linka and Theodoulou 2013). Thus, the peroxisomal bilayer membrane is comparable to the plasma membrane and endoplasmic reticulum (ER) membrane, since permeability is conducted by both, channel and carrier proteins. The combination of non‐selective pore‐forming channels to transfer small molecules as well as specific carrier proteins for ‘bulky’ solutes is a highly efficient way to cope with a massive flux of a variety of metabolites across the peroxisomal border. While solute transport through a pore‐forming channel occurs at a much faster rate, carriers bind their solutes tightly and are thus highly specific to their transport substrate.

**Figure 1:**
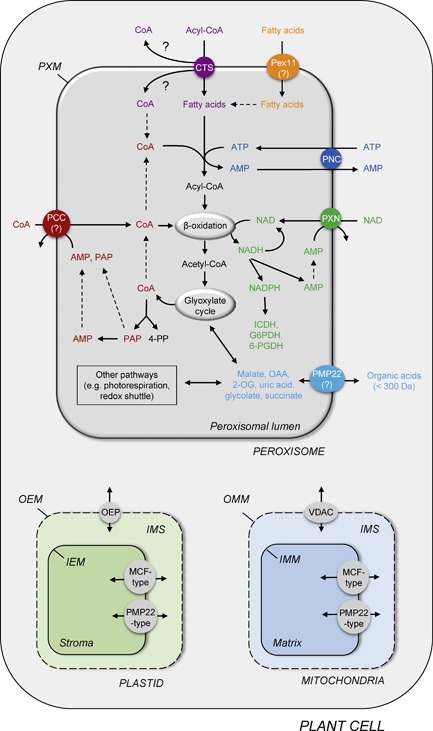
**Scheme of a plant cell presenting the known peroxisomal transporter and channel proteins connecting peroxisomal metabolism with that of the cell** Based on the proposed transport mechanism, the peroxisomal ABC transporter CTS import acyl‐CoA esters into the peroxisomes. During this import process, the substrates is hydrolyzed to fatty acids and CoA and released into the peroxisomal matrix. Such a transport mode requires the intra‐peroxisomal ATP‐dependent re‐esterification to CoA for fatty acid activation. The peroxisomal ATP carrier PNC import cytosolic ATP in exchange with AMP, which is produced during fatty acid activation. ATP is also required for other ATP‐dependent reaction inside peroxisomes, including auxin biosynthesis, protein phosphorylation and mevalonate pathway. NAD‐dependent reactions inside peroxisomes, like fatty acid oxidation, depend on the import of NAD from the cytosol catalyzed by the peroxisomal NAD carrier PXN. The exchange partner for the NAD uptake is generated by peroxisomal NADH hydrolysis. NAD can be phosphorylated to NADPH, which is the cofactor for NADP‐dependent ICDH and the OPPP enzymes G6PDH and 6‐PGDH. In addition, peroxisomes require a peroxisomal CoA carrier (PCC) mediating the uptake of CoA, which has not been identified for plant peroxisomes. If the CoA is cleaved off at the cytosolic side of the peroxisomal membrane during the transport of acyl‐CoA, the peroxisomal CoA carrier imports cytosolic CoA into peroxisomes required for fatty acid activation. Peroxisomal CoA is also used to remove acetyl‐CoA from the fatty acyl chain in the last step of β‐oxidation, whereas the glyoxylate cycle produces free CoA. To regulate CoA homeostasis, CoA can be hydrolyzed to PAP or 4‐PP, which are suitable counter‐exchange substrates for the CoA import. The further hydrolysis of PAP yields AMP, another putative export substrate for the peroxisomal CoA carrier. Other intermediates of the peroxisomal metabolism, such as organic acids, might be shuttled via the peroxisomal channel protein called PMP22 across the peroxisomal membrane. Pex11 as a second channel might be involved in the uptake of free fatty acids into the peroxisomes, as a redundant system to the peroxisomal ABC transporter. Other compartments of a plant cell, like plastids and mitochondria, possess a large variety of transporter and channel proteins for shuttling metabolites across their bilayer membranes, including the outer envelope proteins OEPs, plastidial MCF‐type carriers, plastidial PMP22‐type channels, voltage‐dependent anion channels (VDACs), mitochondrial MCF carriers, and mitochondrial PMP22‐type channels. Abbreviations: PAP, 3’,5’‐ADP; 4‐PP, 4‐Phosphopantetheine; OAA, oxaloacetate; 2‐OG, 2‐oxoglutarate; ICDH, isocitrate dehydrogenase; G6PDH, glucose‐6‐phopshate dehydrogenase; 6‐PGDH, 6‐phosphogluconate dehydrogenase; OEM, outer envelope membrane; IEM, inner envelope membrane; OMM, outer mitochondrial membrane; IMM, inner mitochondrial membrane; IMS, intermembrane space.

Peroxisomal pore‐forming channels are present in the membrane of plants, mammals, and yeast peroxisomes as well as in glycosomal membranes of *Trypanosoma brucei* (Reumann et al. 1995; Reumann et al. 1996; Reumann et al. 1998; Antonenkov et al. 2009; Rokka et al. 2009; Gualdrón‐López et al. 2012; Mindthoff et al. 2015). These channel proteins are general pores consisting of transmembrane α‐helices, and thus, distinct to the β‐barrel porins. The peroxisomal channels allow the rapid and unrestricted diffusion of small hydrophilic molecules with molecular masses up to 300–400 Da, but prevent diffusion of larger molecules across the peroxisomal membrane (Antonenkov and Hiltunen 2012). The transport is driven by the concentration gradient of the transported solute. Recently, two putative channel proteins have been identified for peroxisomes: the peroxisomal membrane protein of 22 kDa (PMP22) and peroxin 11 (PEX11) (Figure 1) (Rokka et al. 2009; Mindthoff et al. 2015).

To date, specific solute transporters belonging to the mitochondrial transporter family (MCF) and the ATP‐binding cassette (ABC) transporter family have been found in the peroxisomal membranes of mammals, plants and yeast (Figure 1) (Linka and Esser 2012; Linka and Theodoulou 2013). These two families of proteins each consist of transmembrane α‐helical domains and catalyze a passive or active transport, respectively. Three peroxisomal members of the MCF are passive carriers mediating the facilitated diffusion of solutes down their electrochemical gradient (Palmieri et al. 2001; van Roermund et al. 2001; Visser et al. 2002; Arai et al. 2008; Linka et al. 2008; Agrimi et al. 2011; Agrimi et al. 2012; Bernhardt et al. 2012). In contrast, the peroxisomal ABC transporter is an active transporter pumping its substrate across the membrane against their electrochemical gradient, which is coupled to the hydrolysis of ATP as energy source (Morita and Imanaka 2012; Baker et al. 2015; Theodoulou and Kerr 2015). In summary, both peroxisomal carrier types perform the traffic of large molecules (up to < 400 Da), including acyl‐CoA esters and some cofactors like ATP, NAD^+^, CoA.

## PEROXISOMAL ABC TRANSPORTERS

The peroxisomal ABC transporters belong to the D subfamily of the ATP‐binding cassette (ABC) transporter family (Morita and Imanaka 2012; Baker et al. 2015; Theodoulou and Kerr 2015). Like all eukaryotic ABC transporters, they have a core structure consisting of two conserved domains: a transmembrane domain (TMD) with multiple α‐helices and a nucleotide‐binding domain (NBD) with Walker A and Walker B motifs. The TMDs bind and translocate substrates, whereas the NBDs form a ‘sandwich dimer’ capable of binding and hydrolyzing ATP. The hydrolysis of ATP provides the energy for the translocation of the substrate against an electrochemical gradient across the membrane (Higgins 1992; Rees et al. 2009). The peroxisomal ABCD proteins from human, mammals and fungi function as dimers of two TMD‐NBD ‘half transporters’ (Morita and Imanaka 2012; Baker et al. 2015; Theodoulou and Kerr 2015). In contrast, plant peroxisomes possess a full‐sized transporter encoded by a single gene. This plant ABCD protein contains two homologous but distinct halves that are attached as heterodimers with the topology TMD1‐NBD1–TMD2‐NBD2 (Dietrich et al. 2009). The fusion of two half transporters into one ‘pseudo‐heterodimeric' protein was a single event in the evolution of the green plant lineage which occurred before the divergence of bryophytes (Morita and Imanaka 2012; Baker et al. 2015; Theodoulou and Kerr 2015). The role of the peroxisomal ABCD proteins has been intensively studied in yeast, human and plants. Cross‐species complementation studies revealed a conserved function for members of the ABCD family, mediating the uptake of β‐oxidation substrates into peroxisomes (Table 1).

**Table 1:** Plant peroxisomal transport proteins described in this review, their predicted transport substrates and transport mode

Transporter	Transport substrates	Transport mode
PNC	ATP_in_/ADP_out_	Passive transport down the chemical gradient in an antiport mechanism
	ATP_in_/AMP_out_	
PXN	NAD_in_/AMP_out_	Passive transport down the chemical gradient in an antiport mechanism
PCC	CoA_in_/AMP_out_	Passive transport down the chemical gradient in an antiport mechanism
	CoA_in_/PAP_out_	
PMP22	Organic acids like Malate, OAA, 2‐OG, glycolate succinate	Passive transport down the chemical gradient in an uniport mechanism
PEX11	long‐chain and medium‐chain fatty acids	Passive transport down the chemical gradient in an uniport mechanism
CTS	Acetyl‐CoA	ATP‐dependent active transport in an uniport mechanism

Yeast contains two peroxisomal half‐ABC transporters ScPxa1p and ScPxa2p (Hettema et al. 1996; Shani and Valle 1996; Verleur et al. 1997). Single knockout mutants are impaired in growth on oleate‐containing medium, indicating that they are unable to utilize oleate (C18:1) as a sole carbon source (Hettema et al. 1996; Verleur et al. 1997). The double yeast mutant *pxa1*Δ/*pxa2*Δ did not display an enhanced phenotype compared to the single mutants, suggesting that the two half‐size transporters heterodimerize to form a fully functional transporter. This was confirmed by protein–protein interaction studies (Shani and Valle 1996; Chuang et al. 2014). The growth defect was restricted to only very‐long chain fatty acids (VLCFA; >C22:0), indicating that ScPxa1p/ScPxa2p is involved in the import of fatty acids with 18 or more carbons as CoA esters into the peroxisome for their further degradation by β‐oxidation (Hettema et al. 1996; Verleur et al. 1997). In yeast an alternative uptake route exists for medium‐chain free fatty acids, which is independent of the peroxisomal ABC transporter (van Roermund et al. 2003). These β‐oxidation substrates can enter the peroxisome in the non‐esterified form via the proposed ScPex11 channel protein (Mindthoff et al. 2015). Inside the peroxisomal matrix, they are activated by the peroxisomal acyl‐CoA synthetase ScFaa2p (fatty acid activation protein 2) (Hettema et al. 1996), prior to β‐oxidation, a process which requires ATP imported by the peroxisomal ATP carrier ScAnt1p (adenine nucleotide transporter1) (Palmieri et al. 2001; van Roermund et al. 2001).

Humans have three ABCD family members residing in the peroxisomal membrane (ABCD1‐3) (Kamijo et al. 1990; Mosser et al. 1993; Lombard‐Platet et al. 1996). They are half transporters and function *in vivo* as active homodimers. The human ABCD proteins have distinct but partially overlapping substrate specificities to different acyl‐CoA esters. HsABCD1 shares functional redundancy with HsABCD2 (van Roermund et al. 2008, 2011). A defect in the human ABCD1 protein (or ALDP) causes X‐linked adrenoleukodystrophy (X‐ALD) (Mosser et al. 1993). This most common peroxisomal disorder is characterized by an impaired peroxisomal β‐oxidation and consequently accumulation of VLCFA in tissues, especially the brain and the adrenal glands (Wiesinger et al. 2013). Although human ABCD proteins have functional redundancy, the basal expression levels of HsABCD2 and HsABCD3 are not sufficient to compensate the lack of HsABCD1 in X‐ALD patients. Human ABCD2 (or ALDR) prefers shorter VLCFA and polyunsaturated fatty acids as transport substrates (van Roermund et al. 2011), whereas human ABCD3 (or PMP70) has the broadest substrate specificity (van Roermund et al. 2014). It is involved in the transport of saturated fatty acids, unsaturated fatty acids, branched‐chain fatty acids, dicarboxylic fatty acids, and C27 bile acid intermediates (Ferdinandusse et al. 2015).

The *Arabidopsis* ABCD1 is the most studied and best understood plant peroxisomal ABC transporter. It has been independently identified by four different groups, and hence is also known as CTS (Comatose [Footitt et al. 2002]), PXA1 (peroxisomal ABC transporter 1 [Zolman et al. 2001]), PED3 (peroxisome defective 3 [Hayashi et al. 2002]) and ACN2 (acetate non‐utilizing 2) (Hooks et al. 2004). In this review, we use the name CTS. *Arabidopsis* plants defective in CTS exhibit different phenotypes with respect to β‐oxidation. Besides fatty acid degradation β‐oxidation is involved in a wide range of metabolic and signaling processes in plants, including the synthesis of signaling molecules and secondary products. Hence, *Arabidopsis* CTS has been implicated not only to import fatty acyl‐CoAs into the peroxisome, but also various other β‐oxidation substrates, such as the jasmonic acid (JA) precursor 12‐oxo‐phytodienoic acid (OPDA) (Theodoulou et al. 2005; Dave et al. 2011), the synthetic auxin precursor 2,4‐dichlorophenoxybutyric acid (2,4‐DB) (Hayashi et al. 2002) and indole butyric acid (IBA) (Zolman et al. 2001), precursors of ubiquinone synthesis (Block et al. 2014) and cinnamic acid (Bussell et al. 2014), which is required for the synthesis of benzoic acid.

An unusual transport mechanism has been proposed for the human and *Arabidopsis* ABCDs shuttling their substrates across the peroxisomal membrane (Figure 1). Acyl‐CoA esters and not ‘free’ (non‐esterified) fatty acids are bound to the human ABCD1‐3 and CTS transporter, demonstrated by the stimulated ATPase activity in the presence of fatty acyl‐CoA derivatives (Nyathi et al. 2010). Once the acyl‐CoA substrate is bound by ABCD or CTS, the CoA moiety is cleaved off during transfer across the lipid bilayer. The CoA cleavage during the transport cycle of CTS was confirmed using isotopic labelling of yeast cells with ^18^O (van Roermund et al. 2012). Furthermore, it was experimentally shown that the human ABCD1‐3 and CTS itself possess an intrinsic acyl‐CoA thioesterase activity (De Marcos‐Lousa et al. 2013; Geillon et al. 2017; Okamoto et al. 2018), although no motif homologous to either α/β‐hydrolases or hot‐dog fold thioesterases were detected (De Marcos‐Lousa et al. 2013). Such a transport mechanism subsequently requires a re‐activation within the peroxisome by a peroxisomal acyl‐CoA synthetase (ACS), which interacts with the transporter (De Marcos‐Lousa et al. 2013). It was shown that functional complementation of the *pxa1*Δ/*pxa2*Δ yeast mutant by CTS depends on the presence of ScFaa2p or the equivalent *Arabidopsis* long‐chain acyl CoA synthetases 6 and/or 7 (LACS6/7) inside yeast peroxisomes (De Marcos‐Lousa et al. 2013). Coupling the translocation process to the peroxisomal ATP‐dependent re‐activation with CoA might be a potential regulation point for the entry of fatty acids and other substrates into the β‐oxidation pathway. The cleavage and re‐esterification of the acyl‐CoA consumes two molecules of ATP and thus seems to be energetically wasteful. Still, it solves the biophysical challenge of shuttling an amphipathic molecule across the peroxisomal membrane and enables separate permeation pathways for the hydrophilic (CoA) and hydrophobic (fatty acid) moieties of β‐oxidation substrates. In addition, the human ABCD and plant CTS transporter are able to accept disparate CoA derivatives as transport molecules via the intrinsic thioesterase domain, which recognizes the CoA moiety as an important determinant. We do not know yet, on which side of the peroxisomal membrane the CoA cleavage by the HsABCD1‐3 or CTS transporter occurs. If CoA is released in the cytosol, the question how it enters peroxisomes remains to be answered.

## PEROXISOMAL MEMBERS OF THE MITOCHONDRIAL CARRIER FAMILY

The mitochondrial carrier family (MCF) represents the largest group of eukaryotic transport proteins with 35 members in *Saccharomyces cerevisiae* (Palmieri et al. 2000, 2006) and more than 50 putative candidates in human (Palmieri 2014). The *Arabidopsis* genome encodes for 58 MCF members evenly distributed across the five chromosomes (Haferkamp 2007; Palmieri et al. 2011; Haferkamp and Schmitz‐Esser 2012). All MCF carriers contain three tandemly repeated homologous domains, each consisting of two hydrophobic membrane spanning α‐helices linked by a conserved sequence motif (Palmieri et al. 2011; Haferkamp and Schmitz‐Esser 2012; Taylor 2017). They are highly variable in terms of size and charge of the transported molecule and the underlying transport mode. The predominant mechanism follows a 1:1 exchange of solutes (antiport), but unidirectional substrate transport (uniport) and proton compensated anion symport have been shown to be mediated by certain MCF carriers (Palmieri et al. 2011; Haferkamp and Schmitz‐Esser 2012; Palmieri 2014; Taylor 2017). According to their substrate specificity, they can be divided into four subfamilies. The first group includes the nucleotide and nucleotide derivate transporters. Carriers for the transport of di‐ and tri‐carboxylates and keto‐acids belong to the second subfamily. The third group comprises carriers catalyzing the transport of amino acids and their derivatives. Other carriers are grouped together with uncoupling proteins in the fourth and last subfamily (Palmieri et al. 2011; Haferkamp and Schmitz‐Esser 2012; Palmieri 2014; Taylor 2017). Despite their name several MCF carriers have been localized to other cellular compartments, such as plasma membrane, ER membrane, plastids and peroxisomes. Up to now, three peroxisomal MCF carriers have been characterized in human, mammals, fungi and plants mediating the transport of ATP, NAD and CoA (Figure 1, Table 1) (Palmieri et al. 2001; van Roermund et al. 2001; Arai et al. 2008; Linka et al. 2008; Agrimi et al. 2011; Agrimi et al. 2012; Bernhardt et al. 2012). These essential cofactors are synthetized outside the peroxisomes, and therefore, have to be imported into the peroxisomal lumen (Figure 1). In all cases a specific carrier is required, because ATP, NAD and CoA cannot be transported through a peroxisomal pore‐forming channel due to their size.

### The peroxisomal ATP carrier

The first peroxisomal MCF carrier found was the ATP carrier from *S. cerevisiae*, called ScAnt1p (Adenine nucleotide transporter 1) (Palmieri et al. 2001; van Roermund et al. 2001). Based on sequence similarity to ScAnt1p, two *Arabidopsis* MCF members have been identified called AtPNC1 and AtPNC2 (Peroxisomal adenine Nucleotide Carrier) (Arai et al. 2008; Linka et al. 2008). Their transport function has been investigated by *in vitro* uptake experiments using liposomes reconstituted with recombinantly expressed proteins (Table 1). The yeast and plant carrier accept the adenine nucleotides ATP, ADP and AMP as transport substrates and catalyze a strict counter exchange of ATP against AMP or ADP (Palmieri et al. 2001; Linka et al. 2008). The import of cytosolic ATP into peroxisomes is essential for the ATP‐dependent activation of fatty acids for their degradation by peroxisomal β‐oxidation (Figure 1) (Palmieri et al. 2001; van Roermund et al. 2001; Arai et al. 2008; Linka et al. 2008). In yeast and plants, the fatty acid β‐oxidation exclusively takes place inside peroxisomes (Goepfert and Poirier 2007; Graham 2008; van Roermund et al. 2012). Consequently, yeast cells deficient in ScAnt1p were unable to metabolize medium‐chain fatty acids, such as lauric acid (C16:0) as carbon and energy source due to the lack of intraperoxisomal ATP (Palmieri et al. 2001; van Roermund et al. 2001). *Arabidopsis* PNC1 and PNC2 were shown to be able to complement the β‐oxidation phenotype of the *ant1*Δ yeast mutant, indicating that the plant carrier, like the yeast ortholog, are able to supply β‐oxidation with ATP (Linka et al. 2008). A similar phenotype was observed for PNC1 and PNC2 *Arabidopsis* mutant plants. While the phenotype of single *pnc Arabidopsis* lines was not distinguishable from the wild type, plants in which both AtPNC proteins are suppressed were impaired in peroxisomal β‐oxidation, leading to a block in storage oil mobilization (Linka et al. 2008). Since storage of carbohydrates (starch) and proteins is minor in *Arabidopsis* seeds (Goepfert and Poirier 2007; Graham 2008), *pnc1/pnc2 Arabidopsis* seedlings depend on exogenous sucrose to allow seedling establishment (Linka et al. 2008). This strongly emphasizes that AtPNC1 and AtPNC2 are the sole site of peroxisomal ATP supply. The fatty acid activation step by peroxisomal acyl‐CoA synthetases produces AMP and PP_i_ in yeast and *Arbaidopsis* (Hettema et al. 1996; Fulda et al. 2004). While AMP is the direct counter‐exchange substrate for the peroxisomal ATP carrier‐mediated ATP import, the destiny of PP_i_ remains unknown. So far, a peroxisomal pyrophosphatase, catalyzing the hydrolysis of PP_i_ to 2 molecules of P_i_ has not been found in peroxisomes. This raises the question how PP_i_ and/or P_i_ are shuttled out of the peroxisomes (Linka and Theodoulou 2013). Due to their polar nature, both solutes are unlikely to freely diffuse across the peroxisomal membrane. Experiments using isolated bovine kidney peroxisomes observed transport activity for P_i_ and to a lesser extent PP_i_ (Visser et al. 2005). Whether specific transporters or unspecific channels mediate this solute transport was not addressed in this study.

Beyond fatty acid β‐oxidation peroxisomal ATP is required for other enzymatic reactions inside peroxisomal lumen. In plants, for example, β‐oxidation is involved in phytohormone biosynthesis (Wasternack and Strnad 2017; Wasternack and Feussner 2018). Jasmonates constitute a family of bioactive oxylipids that regulate a variety of defense responses and developmental processes. Its biosynthesis starts with the production of OPDA and dinor‐OPDA in the chloroplasts and continues in peroxisomes by an alternative β‐oxidation cycle (Wasternack and Strnad 2017; Wasternack and Feussner 2018). The peroxisomal import of OPDA is at least partially mediated by the peroxisomal ABC transporter CTS in *Arabidopsis* (Theodoulou et al. 2005). Inside the peroxisomal matrix it is then reduced to OPC‐8 and activated by peroxisomal AtOPC‐8:CoA ligase (Kienow et al. 2008). *Arabidopsis* null mutants lacking this enzyme are compromised in JA biosynthesis, leading to reduced JA levels (50–60%) and hyperaccumulation of OPC8:0 in wound‐induced leaves (Koo et al. 2006). Finally, the last steps comprise several cycles of β‐oxidation, by which an even number of carbons is removed from the carboxyl side chains of OPC‐8, giving rise to JA in *Arabidopsis*. We also observed in the *pnc1/2 Arabidopsis* silencing mutant significantly reduced JA levels, indicating a role for PNC proteins in JA biosynthesis in plants (personal communication).

In addition to JA, plant peroxisomes are involved in the synthesis of indolce‐3‐acetic acid (IAA), short auxin (Korasick et al. 2013). This phytohormone plays a fundamental role in plant growth and development. It can be stored in inactive forms, conjugated to amino acids or sugars (Korasick et al. 2013). One of several auxin precursors in plants is IBA. Auxin derived from IBA is important during seedling development, when it influences lateral rooting (De Rybel et al. 2012), cotyledon and root hair expansion, and apical hook formation (Strader et al. 2010; Strader et al. 2011). The synthesis of auxin from IBA occurs in plant peroxisomes by the action of an alternative peroxisomal β‐oxidation pathway. Like OPDA and fatty acids, IBA has to be esterified with CoA, an ATP‐consuming reaction in plants. However, in contrast to fatty acid β‐oxidation, an alternative peroxisomal acyl‐CoA synthetase is required, since *Arabidopsis* double knockout mutants of the two *Arabidopsis* fatty acid acyl‐CoA synthetases LACS6/7 are sensitive to IBA (Cassin‐Ross and Hu 2014a). However, even if catalyzed by another enzyme, ATP is still mandatory for the CoA esterification since *pnc1/pnc2 Arabidopsis* mutant plants are less sensitive to IBA as well as to the synthetic auxin precursor 2,4‐DB (Arai et al. 2008; Linka et al. 2008).

Interestingly, there is no obvious phenotype of *Arabidopsis* plants lacking PNC1 and PNC2 after the seedling becomes photoautotrophic (personal communications). Since ATP as the energy currency plays such an important role in the cellular metabolism, it is likely that other peroxisomal pathways in addition to β‐oxidation need ATP. This includes the mevalonate pathway (MVA) in plants that is involved in the formation of isopentenyl diphosphate (IPP) and dimethylallyl pyrophosphate (DMAPP), the building blocks of isoprenoids (Simkin et al. 2011; Rodríguez‐Concepción and Boronat 2015). In *Arabidopsis*, two ATP‐dependent enzymes of the mevalonate pathway (MVA) are located to peroxisomes: phosphomevalonate kinase (PMK) and mevalonate diphosphate decarboxylase (MVD) (Sapir‐Mir et al. 2008; Simkin et al. 2011). AtPMK catalyzes the phosphorylation of mevalonate phosphate to mevalonate diphosphate, consuming ATP and releasing ADP. Mevalonate diphosphate is subsequently decarboxylated by AtMVD to IPP, coupled to the hydrolysis of ATP to ADP and P_i_ (Simkin et al. 2011). IPP derived from the MVA pathway is required in plants for the formation of triterpenes, sesquiterpenes, phytosterols, ubiquinone, vitamin D and primary metabolites important for cell integrity (Rodríguez‐Concepción and Boronat 2015). In parallel to the MVA, IPP can be produced by the plastidic 2‐C‐methyl‐D‐erythritol‐4‐phosphate (MEP) pathway in plants, which is used as a precursor for the synthesis of monoterpenes, carotenoids, apocarotenoids and the side chain of chlorophylls, tocopherols and prenylquinones (Rodríguez‐Concepción and Boronat 2015). Interaction between MVA and MEP pathways has been documented, explaining the less prominent phenotype of single mutants in either of the two pathways (Hemmerlin et al. 2012). However, the plant‐specific carrier and channel involved in peroxisomal and plastidic transport steps have not been identified, yet (Linka and Theodoulou 2013). Since *Arabidopsis* PNCs are the sole source of peroxisomal ATP, *pnc1/2* mutant plants should be affected in MVA‐synthesized isoprenoid molecules, like sterols and volatiles as it was shown for *mvd* mutant plants (Henry et al. 2015). However, a severe phenotype for *Arabidopsis pnc1/2* mutants might not be visible under normal conditions due to the presence of the functional MEP pathway.

Reversible post‐translational modification by phosphorylation is an essential fast responding regulatory mechanism that controls many cellular processes (Cohen 2000; Friso and van Wijk 2015). For this, protein kinases transfer the γ‐phosphate group from ATP to the hydroxyl group of serine, threonine or tyrosine residues, whereas protein phosphatases hydrolyze the phosphoester bond to dephosphorylate proteins. In plants, several soluble peroxisomal proteins involved in β‐oxidation, glyoxylate cycle and photorespiration have been shown to be phosphorylated under certain conditions (Hodges et al. 2013; Kataya et al. 2015). But the physiological effects are still unknown. While many kinases have been identified and characterized, the knowledge about peroxisomal kinases is still limited. The first identified peroxisomal kinase was glyoxysomal protein kinase 1 (GPK1) in *Arabidopsis* (Fukao et al. 2003). AtGPK1 is a serine/threonine protein kinase, most likely anchored to the peroxisomal membrane with its kinase domain facing the peroxisomal lumen. The targets of AtGPK1 have not been identified, but regulation of proteins involved in fatty acid degradation may be required to prevent an overproduction of sucrose and hence protect against waste of energy in early post‐germinative seedlings. In addition, *Arabidopsis* calcium‐dependent protein kinase 1 (CDPK1) has been localized to peroxisomes and oil bodies and has been reported to be involved in salt and drought stress response as well as pathogen resistance (Coca and San Segundo 2010). It is suggested to play a role in lipid metabolism in plants since *Arabidopsis* mutants are partially resistant to the synthetic auxin precursor 2,4‐DB and OPDA (Cassin‐Ross and Hu 2014b). Further research is required to determine the signal transduction pathway of AtCDPK1. Although the *Arabidopsis pnc1/2* silencing plants develop normally through the plant life cycle, it might be interesting to investigate, to what extent the peroxisomal phospho‐proteome was altered in these plant mutant lines.

NAD and its phosphorylated analog (NADP) have become well established as key energy transducers (Pollak et al. 2007; Houtkooper et al. 2010). The *Arabidopsis* genome harbors about 800 predicted oxidoreductases, which are likely to use NAD or NADP as cofactors. They play a fundamental role in reduction/oxidation (redox) metabolism through their contribution to the redox status of compounds, such as glutathione, thioredoxins and ascorbate (Noctor and Foyer 2016). In plant peroxisomes NADP‐depending reactions are catalyzed by enzymes of the oxidative pentose phosphate pathway, such as glucose‐6‐phosphate dehydrogenase and 6‐phosphogluconate dehydrogenase, but also the peroxisomal isocitrate dehydrogenase depends on NADP (Meyer et al. 2011; Hölscher et al. 2014, 2016). All three enzymes are key components of the defense machinery against oxidative stress in plants. The only pathway for *de novo* synthesis of NADP and NADPH is the ATP‐dependent phosphorylation of NAD and NADH, respectively. This reaction is catalyzed in *Arabidopsis* by members of the NAD(H) kinase family ATNADK1, AtNADK2 and AtNADK3. While AtNADK1 and AtNADK2 localize to the cytosol and plastids, respectively, AtNADK3 was shown to reside in peroxisomes (Turner et al. 2004, 2005; Chai et al. 2006; Waller et al. 2010). In contrast to AtNADK1 and AtNADK2, AtNADK3 phosphorylates NADH, but has a lower affinity to NAD (Turner et al. 2005). This might prevent the intraperoxisomal phosphorylation of NAD, which is required for oxidative reactions like β‐oxidation. NADPH produced by *Arabidopsis* NADK3 is not only used for the detoxification of the concomitantly generated H_2_O_2_ in the peroxisomal matrix. It is also required in plants to reduce unsaturated fatty acids (Behrends et al. 1988; Gurvitz et al. 1997; Hua et al. 2012) and the JA precursor OPDA for their conversion via β‐oxidation (Schaller et al. 2000; Stintzi and Browse 2000). The depletion of peroxisomal NADP(H) in case of the *Arabidopsis nadk3* knockout led to impaired biotic and abiotic stress responses either directly linked to pathogen resistance or as part of the peroxisomal antioxidant machinery (Chai et al. 2006; Waller et al. 2010).

All of the above described peroxisomal reactions depend on the import of cytosolic ATP, since plant peroxisomes are unable to synthesize ATP by substrate‐level phosphorylation. As AtPNC1 and AtPNC2 are up to now the only known peroxisomal ATP import route in *Arabidopsis*, *pnc1/2* silencing plants should be deficient in more processes than just fatty acid β‐oxidation. Thus, the generation of *Arabidopsis* null mutants for both PNC1 and PNC2, if vital, are required to explore the impact of ATP deficiency for other ATP‐dependent processes in plant peroxisomes.

### The peroxisomal NAD carrier

Peroxisomes require a specific carrier that mediates the import of NAD into the peroxisomal matrix to supply numerous peroxisomal redox reactions inside peroxisomes (Figure 1) (Noctor et al. 2006; Gakière et al. 2018). In plants, peroxisomal pathways, such as β‐oxidation, photorespiration and ROS detoxification, are essential for peroxisome function and strictly require NAD as cofactor (Hu et al. 2012). To maintain the flux through these metabolic pathways, regeneration of reducing equivalents needs to be assured (Linka and Esser 2012; Linka and Theodoulou 2013). It is widely accepted that the peroxisomal malate/oxaloacetate shuttle is essential for the indirect exchange of the oxidized and reduced forms of NAD in plant peroxisomes (Pracharoenwattana et al. 2007, 2010). The peroxisomal malate dehydrogenase, which is part of this shuttle, produces either NAD or NADH by catalyzing the reversible reduction of oxaloacetate to malate. These dicarboxylates are exported to the cytosol for the re‐conversion by cytosolic malate dehydrogenase and re‐imported into peroxisomes via peroxisomal pore‐forming channels (Antonenkov and Hiltunen 2012).

Since the *de novo* biosynthesis of NAD as well as salvage pathways are located in the cytosol in plants, NAD has to be imported into the peroxisomal lumen (Noctor et al. 2006; Hashida et al. 2009). A peroxisomal NAD carrier has been discovered in *Arabidopsis* (Agrimi et al. 2012; Bernhardt et al. 2012). This *Arabidopsis* carrier, called PXN, catalyzes the transport of NAD, NADH, AMP, ADP and CoA in a strict exchange mode *in vitro* using a liposome uptake system (Table 1) (Agrimi et al. 2012; Bernhardt et al. 2012). In contrast to previously characterized plastidial and mitochondrial NAD antiporters, AtPXN accepts NADH and CoA as substrates. Concentration‐dependent CoA uptake experiments demonstrated that AtPXN had a lower affinity to CoA, catalyzing only marginal CoA uptake into liposomes even at high CoA concentrations (van Roermund et al. 2016). Since the cellular CoA levels are rather low in plants, a physiological role of AtPXN in supplying peroxisomes with CoA is unlikely. To elucidate whether AtPXN mediates the exchange of NAD/AMP or NAD/NADH in a living system, selected *S. cerevisiae* mutants were used (van Roermund et al. 2016). A yeast strain lacking the peroxisomal malate dehydrogenase 3 (ScMdh3p) led to yeast cells that were unable to metabolize fatty acids via peroxisomal β‐oxidation, because NAD is completely reduced to NADH and cannot be re‐oxidized (van Roermund et al. 1995). Under this condition the peroxisomal NADH pyrophosphatase 1 (Npy1p) from *S. cerevisiae* hydrolyzes NADH to AMP, preventing the accumulation of NADH in the peroxisomal matrix (AbdelRaheim et al. 2001). Thus, in this mutant background mainly AMP would be available for AtPXN as internal substrate for the uptake of NAD into yeast peroxisomes. Furthermore, the gene encoding for ScNpy1p was deleted in the *mdh3*Δ yeast background (van Roermund et al. 2016). It was expected that the elevated NADH pool in the peroxisomes of this double mutant supports the NAD import against peroxisomal NADH, mediated by the overexpressed AtPXN. However, AtPXN was able to enhance the fatty acid degradation activity in the *mdh3*Δ yeast mutant, but not in the *mdh3/npy1*Δ yeast double mutant, indicating that the function of ScNpy1p is crucial for phenotype suppression (van Roermund et al. 2016). It is assumed that AtPXN catalyzes the influx of NAD into yeast peroxisomes versus AMP *in vivo*. As a consequence, how is net influx of NAD into peroxisomes guaranteed via such an antiport mechanism? To balance the loss of peroxisomal AMP, cytosolic AMP is re‐imported into peroxisomes by an unknown peroxisomal carrier. An adenylate uniporter could refill the peroxisomal adenine nucleotide pool. In contrast, AMP as counter‐exchange substrate for the NAD import is provided by the hydrolysis of NADH mediated by the peroxisomal nudix hydrolase NUDT19 in *Arabidopsis* (Ogawa et al. 2005, 2008; Reumann et al. 2009). The recombinant protein exhibits NADH hydrolysis activity, and thus is a promising candidate catalyzing this enzymatic step.

To investigate whether *Arabidopsis* PXN provides peroxisomal reactions with NAD in plants, a loss‐of‐function would affect the action of NAD‐dependent pathways, such as β‐oxidation, photorespiration and ROS detoxification. Indeed, the mobilization rate of seed‐stored fatty acids via β‐oxidation is impaired in *Arabidopsis pxn* knockout plants (Bernhardt et al. 2012). However, the degradation of fatty acid is not completely blocked, allowing a normal seedling establishment. The *Arabidopsis* T‐DNA insertion lines for AtPXN displayed an obvious affect during plant development under standard growth conditions (personal communications), indicating that other peroxisomal NAD‐dependent reactions are not restricted in plants. A photometric screen discovered a possible contribution of AtPXN to photorespiration under fluctuating and high light conditions (Li et al. 2018). This weak *Arabidopsis pxn* phenotype suggests alternative routes for the NAD uptake in plant peroxisomes (Bernhardt et al. 2012). Most likely a redundant carrier takes over the function of AtPXN in importing NAD into the plant peroxisomal matrix.

### The peroxisomal CoA carrier

CoA is an essential acyl group carrier and carbonyl‐activating group and it is utilized in the biosynthesis and catabolism of both primary and secondary metabolites; in plants, for example, for the citric acid cycle (mitochondria), fatty acid biosynthesis (plastids) and degradation (peroxisomes) (Coxon et al. 2005). The CoA biosynthesis is mainly localized to the cytosol in eukaryotes and starts with the condensation of pantoate to β‐alanine to form pantothenate (vitamin B5) catalyzed by pantothenate synthase. Subsequent enzymatic steps lead to the production of dephospho‐CoA that is finally phosphorylated by dephospho‐CoA kinase to produce CoA (Rubio et al. 2006; Tilton et al. 2006; Rubio et al. 2008). To supply CoA dependent metabolic reactions in different organelles (cell compartments), the transfer of CoA across biological membranes is essential (Linka and Esser 2012; Linka and Theodoulou 2013). Members of the MCF have been shown to mediate this shuttle for an efficient subcellular distribution of this cofactor within the eukaryotic cell (Figure 1).

Leu5p and SLC25A42 represent mitochondrial CoA carriers in yeast and human, respectively (Prohl et al. 2001; Fiermonte et al. 2009). Based on sequence similarity, both mitochondrial CoA carriers from *Arabidopsis* AtCoAC1 and AtCoAC2 and their maize homologs have been identified (Zallot et al. 2013). All four proteins were able to functionally complement a ScLeu5 deficient yeast strain (Zallot et al. 2013). Yeast cells lacking LEU5 display retarded growth on rich media containing a non‐fermentable carbon source, such as glycerol, and the CoA levels in intact *leu5*Δ mitochondria were strongly reduced (Prohl et al. 2001). Transport measurements in liposomes reconstituted with *E. coli* expressed human CoA carrier SLC25A42 demonstrated that this carrier transports, besides CoA, dephospho‐CoA, adenosine 3’,5’‐diphosphate (PAP), and the adenine nucleotides by counter‐exchange (Fiermonte et al. 2009).

In plants though, a peroxisomal CoA carrier (PCC) is still unknown (Figure 1). The peroxisomal NAD carrier PXN from *Arabidopsis* showed transport activities for CoA *in vitro* (Agrimi et al. 2012), but such a CoA import function is unlikely to occur under physiological conditions (van Roermund et al. 2016). The only peroxisomal CoA carrier, characterized so far, is the human MCF member SLC25A17, which is also known as ANT1 or PMP34 (Agrimi et al. 2011). *In vitro* uptake assays revealed that the recombinant SLC25A17 protein − as its mitochondrial counterpart − also exhibits a broad substrate specificity (Agrimi et al. 2012). Notably, the human SLC25A17 is able to catalyze the flux of intermediates of the CoA biosynthesis and salvage pathway. In *Arabidopsis*, proteomic data imply that dephospho‐CoA kinase is localized to peroxisomes, plastids and the cytosol (Reumann et al. 2009), thus giving the possibility that dephospho‐CoA is transported in addition or instead of CoA. Transport function for PAP by the human SLC25A17 carrier gives rise to a potential CoA salvage pathway within peroxisomes in general. The yeast peroxisomal Pcd1p is a nudix hydrolase with specificity to CoA producing PAP and 4’‐phosphopantetheine (4‐PP) (Cartwright et al. 2000). NUDT7a and Y87G2A.14, the Pcd1p homologs in mouse and *C. elegans*, respectively, also show CoA hydrolase activity (Gasmi and McLennan 2001; AbdelRaheim and McLennan 2002; Reilly et al. 2008). While in *Arabidopsis* CoA hydrolysis activity of AtNUDT19 is almost undetectable (Ogawa et al. 2005, 2008), NUDT15 might be a better candidate catalyzing this reaction in *Arabidopsis* (Ito et al. 2012). AtNUDT15 has a splice variant NUDT15a with an early stop codon unveiling a putative peroxisomal targeting signal. Both variants have an N‐terminal mitochondrial target peptide. Masking this domain results in a peroxisomal localization (Ito et al. 2012). The product of the CoA hydrolysis (PAP and 4‐PP) might be used as counter‐exchange substrates for the CoA import. 4‐PP as a CoA precursor can then enter CoA biosynthesis pathway in the cytosol. Putative transport function of 4‐PP by the peroxisomal CoA carrier in plants needs to be tested *in vitro*. As an alternative fate for PAP inside peroxisomes, it could be converted to AMP by an unknown peroxisomal hydrolase. The resulted AMP might function as exchange partner for CoA or NAD import (Table 1).

As a reminder, for the peroxisomal ABC transporter in *Arabidopsis* CTS it was demonstrated that during the transport process of acyl‐CoA esters the CoA moiety is cleaved off. If the CoA molecule is released into the peroxisomal matrix, the need for a peroxisomal CoA importer would obliviate. In this scenario, the role of a peroxisomal CoA carrier in plants could be to regulate the peroxisomal CoA homeostasis by exporting CoA from the peroxisomes. For instance, in plants each glyoxylate cycle releases free CoA into the peroxisomal lumen. Still, due to the presence of the human peroxisomal CoA carrier it is most likely that in plants the CoA carrier is also a member of the MCF.

## PEROXISOMAL PORE‐FORMING CHANNELS

Several reports provided evidence on pore‐forming channels in membranes of peroxisomes from human, mammals, plant, yeast and *Trypanosoma brucei*, indicating that the existence of these channel proteins is highly conserved within the eukaryote lineage (Reumann et al. 1995, 1996, 1998; Antonenkov et al. 2009; Rokka et al. 2009; Gualdrón‐López et al. 2012; Mindthoff et al. 2015). The detected channel activities in the peroxisomal membrane were studied using the lipid bilayer technique (Andreoli 1974; Ehrlich 1992). Integral proteins were detergent‐solubilized from peroxisomal membranes and incorporated into the planar lipid bilayer (also called ‘black membrane’) which is formed over an aperture located in a septum separating two chambers. Each chamber is filled with buffered ionic solution, for example, with potassium chloride (KCl) solution as electrolyte (Andreoli 1974; Ehrlich 1992). This experimental set‐up allows to record the facilitated diffusion of ions or various organic anions through the channel‐forming pore. For a detailed overview of the lipid bilayer measurements describing peroxisomal channel activities we refer the reader to a review by Antonenkov and Hiltunen (2012).

Electrophysiological studies revealed that the peroxisomal channels mediate the transfer of solutes with a broad substrate specificity through the peroxisomal bilayer membrane. In different plant species, such as spinach and castor bean, only one peroxisomal channel protein with comparable characteristics has been investigated (Reumann et al. 1995, 1996, 1997, 1998), whereas in other eukaryotes, such as mouse and yeast, more than one type of channel‐forming proteins with distinct properties has been detected (Antonenkov et al. 2005; Antonenkov et al. 2009; Grunau et al. 2009; Gualdrón‐López et al. 2012). In general, the activities of the peroxisomal channels from different eukaryotes are comparable to each other, but they differ in their properties from known β‐barrel channels, *e.g*. porins of the Gram‐negative bacteria, voltage‐dependent anion channel of the outer mitochondrial membrane (VDAC) and the outer envelope proteins of plastids (OEPs) (Zeth and Thein 2010). Candidates responsible of these peroxisomal pore‐forming activities have been recently identified, such as PMP22 and PEX11, which are described in the next chapter.

### The peroxisomal membrane protein of 22 kDa (PMP22)

The peroxisomal membrane protein of 22 kDa (PMP22) is one of the most abundant integral peroxisomal membrane proteins found in eukaryotes. It belongs to a small family of putative uncharacterized transport proteins, called MPV17/PMP22 family (Saier et al. 2009). This class of channels consists of two subgroups. One subgroup includes members that are found in mitochondria, named after the inner mitochondrial membrane in human (MPV17) (Spinazzola et al. 2006), whereas the other subdivision contains peroxisomal members, like PMP22. Proteins of the MPV17/PMP22 family are multi‐spanning membrane proteins with four putative *α*‐helical transmembrane domains. They contain a conserved protein motif at the C‐terminus (PF04117) and are predicted to form homotrimers. Several reports demonstrated that both the mitochondrial and peroxisomal members of this family are non‐selective channels with similar basic properties (Rokka et al. 2009; Reinhold et al. 2012; Antonenkov et al. 2015). Since the MPV17/PMP22 members do not share sequence or structural similarities with known porin proteins or other channels, they represent a novel channel‐type. Best‐known mitochondrial members of this family are the mitochondrial inner membrane protein MPV17 in human (Spinazzola et al. 2006) and the stress‐inducible yeast MPV17 homolog Sym1 (Trott and Morano 2004; Dallabona et al. 2010). The PMP22 members described so far are the peroxisomal membrane protein 2 in mouse (Pxmp2; Rokka et al. 2009; Vapola et al. 2014) and the peroxisomal membrane protein 22 kDa in *Arabidopsis* (PMP22) (Tugal et al. 1999; Murphy et al. 2003).

The channel activities of the human MPV17 and yeast Sym1p were investigated by lipid bilayer experiments using recombinant proteins (Reinhold et al. 2012; Antonenkov et al. 2015). Both channels form a membrane pore with a similar diameter (1.6–1.8 nm). The size of the pore is large enough to facilitate the transmembrane diffusion of nearly all mitochondrial solutes, including inorganic ions, different metabolites, and even ATP molecules. These non‐selective channels were constitutively open at low and moderate voltages, but they are in the closed conformation at high voltages (±100 mV) (Reinhold et al. 2012; Antonenkov et al. 2015). In case of the peroxisomal members of this MVP17/PMP22 channel family, the channel function of the mouse Pxmp2 protein has been verified (Rokka et al. 2009; Vapola et al. 2014). The recombinant protein also forms a relatively wide, water‐filled pore with an estimated size diameter of 1.4 nm in an artificial lipid bilayer, allowing the passive diffusion of various organic acids with molecular masses up to 300 Da, such as glycolate, pyruvate, and 2‐ketoglutarate (Table 1) (Rokka et al. 2009; Vapola et al. 2014). The basic characteristics of the channel activities of the recombinant Pxmp2 protein are comparable to those measured for the peroxisomal membrane preparations purified from mouse liver (Rokka et al. 2009; Vapola et al. 2014).

In *Arabidopsis* the peroxisomal member of this channel family, called PMP22, was identified as one of the first plant peroxisomal membrane proteins by subcellular fractionation (Tugal et al. 1999). Later immunofluorescence imaging (Murphy et al. 2003) and experimental proteomics (Eubel et al. 2008; Reumann et al. 2009) confirmed this finding. However, whether the *Arabidopsis* PMP22 functions as pore‐forming channel, like the mouse Pxmp2 and contribute to the permeability of plant peroxisomes is so far unknown (Figure 1). Both proteins are members of the same transport protein family and share 30% identity on protein sequence level (Saier et al. 2009).

### The Peroxin 11 (PEX11)

Peroxin 11 (PEX11) is an integral membrane protein of the peroxisomal bilayer membrane with at least two predicted α‐helical transmembrane domains and both termini exposed to the cytosol (Thoms and Erdmann 2005). PEX11 is considered a key player in peroxisomal proliferation, regulating peroxisome size and number in all eukaryotic organisms. The PEX11 protein family consists of three members in human and mammals (Pex11α, Pex11β, and Pex11γ), five members in *Arabidopsis* (PEXa, PEXb, PEXc, PEXd, and PEXe), and three members in yeast (Pex11, Pex25, and Pex27) (Abe and Fujiki 1998; Rottensteiner et al. 2003; Tanaka et al. 2003; Lingard and Trelease 2006). Unexpectedly, the yeast Pex11p also represents a non‐selective channel responsible for transfer of solutes across peroxisomal membrane.

A small part of the PEX11 proteins (∼100 aa) shares 40% sequence similarity to the transient receptor potential cation‐selective channel (Mindthoff et al. 2015). Due to its relationship to the TRP channel family, the yeast PEX11 protein was recombinantly expressed and reconstituted into lipid bilayer to assess channel properties (Mindthoff et al. 2015). The electrophysiological experiments clearly demonstrated that ScPex11p exhibits channel‐forming activities. A pore‐diameter of around 0.6 nm was calculated, implying that molecules with up to 400 Da can pass the non‐selective channel (Mindthoff et al. 2015). Comparative multi‐channel analysis of Pex11‐deficient yeast peroxisomes revealed pore‐forming activities with different characteristics to those of the recombinant ScPex11p, indicating that the yeast peroxisomes possess more than one pore‐forming channel (Mindthoff et al. 2015).

Ectopic overexpression of proteins of the PEX11 family from yeast, plants, mammals or humans induces peroxisome proliferation, leading to the formation of elongated peroxisomes (Abe and Fujiki 1998; Li and Gould 2002; Lingard and Trelease 2006; Nito et al. 2007; Orth et al. 2007; Koch et al. 2010). The deletion of PEX11 in yeast, however, results in fewer but giant peroxisomes in yeast (Erdmann and Blobel 1995). *Arabidopsis* has five paralogs of PEX11, which were subdivided into group 1 (AtPEX11a and AtPEX11b) and group 2 (AtPEX11c, ATPEX11d, and ATPEX11e). In *Arabidopsis* mutant plants in which single PEX11 genes were silenced the peroxisome number and size were reduced (Orth et al. 2007), whereas double and triple *Arabidopsis* knockdown mutants *pex11a/pex11b* and *pex11c/pex11d/pex11e* contain large peroxisomes in the cells of roots and leaves (Nito et al. 2007). Peroxisome proliferation is a multistep process including elongation, constriction and fission (Schrader et al. 2016). Based on the altered peroxisome morphology, it has been implicated that PEX11 proteins directly participate in the first two steps. It enables membrane elongation by stabilizing peroxisomal membrane tubules and thus initiates the formation of constriction sites by recruiting the mitochondrial fission machinery to peroxisomes (Schrader et al. 2016).

Beside the peroxisome proliferation phenotype, yeast cells lacking Pex11p are unable to metabolize long‐ and medium‐chain fatty acids, such as oleic acid (C18:1) and lauric acid (C12:0), as sole carbon and energy source (Table 1) (van Roermund et al. 2000). While the overexpression of ScPex11p led to enhanced β‐oxidation activities in intact yeast cells (Mindthoff et al. 2015). This leads to the question how the peroxisomal fatty acid degradation is linked to the supposed role of PEX11. Changes in PEX11 as an essential regulator of peroxisome proliferation might indirectly lead to dysfunctional peroxisomes with an impaired metabolism. Alternatively, PEX11 as a solute channel mediating the import of medium‐ and long‐chain fatty acids into peroxisomes, supplies peroxisomal β‐oxidation with its substrates. The peroxisomal ABC transporter is essential for the import of very long‐chain fatty acids, but the absence of a pore‐forming protein might alter concentration and composition of solutes inside the peroxisomal lumen. This in turn could negatively influence the process of peroxisome proliferation. Enlarged peroxisomes have been reported for several *Arabidopsis* β‐oxidation mutants, such as *kat2* (β‐Ketothiolase; Germain et al. 2001), *mfp2* (Multifunctional protein; Rylott et al. 2006), and *pxn* (peroxisomal NAD carrier; Mano et al. 2011), assuming that a defective fatty acid degradation indirectly disturbs peroxisome proliferation.

Since other PEX11 orthologs from plants, mammals and humans are able to restore the inability of the *pex11*Δ yeast mutant to grow on oleic acid and rescue the peroxisome morphology phenotype, it remains to be solved how PEX11 proteins can fulfill two distinct functions in the eukaryotic cell − as a solute channel and as a factor required for peroxisome proliferation (Figure 1).

## CONCLUSION

Peroxisomes have to exchange intermediates with other cell compartments to fulfill their role in cellular metabolism. For this functional interplay they rely on the peroxisomal transporters and channels described in this review. For a high‐flux of solutes between two organelles, the membranes of both compartments need also to be in close proximity. Otherwise the slow diffusion of solutes to their cellular destination would rate‐limit the metabolic flux through this pathway. Recently, protein complexes have been discovered that physically connect peroxisomes to mitochondria, for example, via membrane contact sites (MCSs) (Schrader et al. 2015). These membrane regions of tethered organelles are beneficial for rapid solute channeling due to an enrichment of specific transporters and channels and the generation of concentration gradients of solutes at these sites. Further insights into the peroxisomal solute transport processes in respect to specificity and regulation, would be a great step towards using peroxisomes as synthetic organelles for heterologous pathway compartmentalization (Kessel‐Vigelius et al. 2013).

An alternative mechanism for peroxisomes to exchange solutes is the transfer of vesicles from the ER or mitochondria, which both play an important role in peroxisome biogenesis. Peroxisomes derive *de novo* from vesicles from the ER and mitochondria and replicate by growth and division (Sugiura et al. 2017). Therefore, such a vesicular transport might provide peroxisomes with membrane proteins and enzymes, but also with lipids and other metabolites and cofactors.
